# Nourish to flourish: sustainable diets, sports nutrition, and psychology in college life

**DOI:** 10.3389/fnut.2026.1828769

**Published:** 2026-05-13

**Authors:** Jun Huang, Zhuozhi Qi

**Affiliations:** 1 College of Competitive Sports, Shandong Sport University, Rizhao, Shandong, China; 2 Russian State-Run Sports University, Moscow, Russia

**Keywords:** dietary habits, mental health, sports participation, sustainable diet, sustainable food, university students

## Abstract

**Background:**

In this paper, the author examines the intersections of sustainable diets, sports nutrition, and psychology as applied to college life. Since college students experience specific difficulties in academic achievement, physical health, and mental health, this study explores the impact of nutrition and psychological factors on their everyday lives.

**Objective:**

It focuses on sustainable eating habits that not only enhance the environment and long-term health but also the effects of appropriate sports nutrition on academic achievement and physical fitness. Furthermore, the paper explores the psychological side of eating habits, such as body image, stress, and eating behaviors, and emphasizes the significance of mental state in achieving optimal nutrition.

**Methods:**

This study combines the lenses of nutrition science, psychology, and environmental sustainability to provide actionable guidance that helps college students make informed, healthy, and sustainable dietary decisions to improve their bodies and minds.

**Results:**

This paper discusses how sustainable diets affect long-term health outcomes and academic and physical performance in sports nutrition. It also addresses the psychological factors that influence eating habits and their connections to mental health and body image.

**Conclusion:**

The paper highlights the significance of psychological conditions in realizing optimal nutrition and offers practical information to college students to create informed, healthy and sustainable food choices to improve physical and mental health.

## Graphical Abstract

**Figure:**
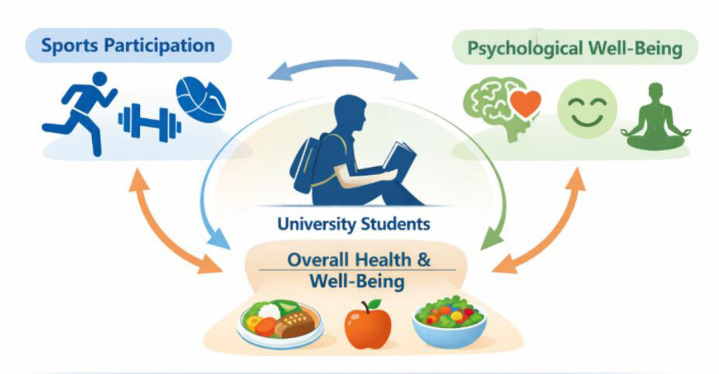

## 1 Introduction

College life is a critical period when young adults must navigate significant changes while balancing academic obligations, community involvement, and personal health (1). Nutrition is one of the primary factors in ensuring students' physical and mental wellbeing. Nevertheless, due to hectic schedules, the stress of college life, and changing lifestyles, most college students find it difficult to make wise, sustainable food choices (2). This paper examines the overlap among sustainable eating, sports nutrition, and psychology through the lens of college life, focusing on how these components affect students' overall health, wellbeing, and academic performance. Sustainable diets, which emphasize both individual and environmental health, are becoming an important part of contemporary dietary practices. Such diets aim to minimize the environmental impacts of food decisions in favor of plant-based, domestically grown, and minimally processed foods (3). Nevertheless, for economic or time reasons, or out of ignorance, so many students still use convenience foods, which are usually low in nutrients and not environmentally friendly. This paper explores the potential benefits of adopting more sustainable eating patterns among the college population, aiming to align environmental awareness with health-related decisions (4). At the same time, sports nutrition is an important factor in enhancing physical performance, particularly among students who engage in sports or other physical activities. A proper diet boosts energy, quickens recovery, and promotes fitness, which is crucial for students who combine physical activities with strenuous academic programs (5). The paper also examines how specific nutrients and supplements can boost athletic performance and how sports nutrition can help students achieve their academic and physical goals. Beyond the physical aspects of nutrition, psychological factors also play an important role in dietary patterns and preferences (6). Emotional eating, body concerns, and stress are common factors that influence how students think about food. Stresses of college life, such as academic pressures and social dynamics, may worsen poor eating habits, including overeating, skipping meals, or reaching for comfort foods. The paper also explores the effect of psychological health on food choices, justifying the relevance of mental health in maintaining a healthy diet (7). By understanding the intricate interplay between psychological wellbeing and nutrition, this paper offers guidance on promoting mental wellbeing through balanced, sustainable eating habits (8). Finally, the objective of the study is to provide in-depth insight into the intersection of sustainable diets, sports nutrition, and psychological determinants that influence the health and academic life of college students. Across the interrelated areas, the paper offers viable solutions to foster healthier, more sustainable food preferences and improve students' general health. This exploration helps make meaningful contributions to students, educators, and policymakers who seek to improve the health of the college community.

## 2 Methodology

The present research uses a multidimensional approach to explore the overlaps among sustainable diets, sports nutrition, and psychology in the lives of college students. Given the complexity of the topic, a qualitative and experimental study design was employed to provide a comprehensive view of the impact of these factors on the health, academic achievement, and wellbeing of college students. The methodology entails an in-depth literature review, a sequence of dietary interventions in a group of students, physical performance tests and psychological profiling. The general objective is to assess how nutritional knowledge and dietary habits affect physical and psychological health, while also considering sustainable, environmentally friendly dietary practices (9). The methodological framework is presented in the following steps:

The literature review was conducted in detail to develop background knowledge of the three most important areas: sustainable diets, sports nutrition, and college students' psychology. This review focused on scholarly articles, clinical studies, and other pertinent reports from reputable organizations.

(i) Sustainable diets
  An exploration of the effects of plant-based diets, local sourcing, and their environmental consequences, their health advantages and problems in the college setting. This is an in-depth discussion of the importance of nutrition in improving athletes' performance, energy-saving techniques, recovery methods, and how students can use nutrition to supplement their physical efforts. How body image concerns, eating stress, emotional eating, and the impact of mental health on eating behaviors among college students (10). The review not only provided the study with context but also helped identify gaps in the current research that the study would address.

### 2.1 Dietary intervention program

A group of 30 college students (male and female) was selected to participate in a dietary intervention program to enhance their understanding of sustainable eating and sports nutrition. The sample was diverse in terms of academic disciplines, as the students were recruited from different fields. This intervention was conducted during 8 weeks and involved two parts: Educational Workshops.

The students took part in weekly workshops where the following topics were discussed: the principles of sustainable diets, the role of nutrition in sports and the psychological aspects of eating. Nutritionists, psychologists, and fitness experts led these workshops, designed to inform students and help them make more sustainable, healthy food choices. Students were asked to record their daily food intake using a food diary app, focusing on meal composition, frequency, and portion size. They also urged them to make changes toward more sustainable eating, including eating more plant-based foods, reducing food waste, and eating locally. Personalized feedback on students' food choices was provided at the end of each week, and their progress was monitored through nutritional assessments. The dietary intervention sought to evaluate the quality of teaching and feedback (one-on-one) in enhancing the knowledge and behavior among students with respect to sustainable eating and sports nutrition.

### 2.2 Physical performance assessments

To determine the effect of dietary changes on physical performance, all individuals underwent pre- and post-intervention physical fitness tests. These included: cardiovascular health.

Students ran for 12 min to measure cardiovascular endurance, and the distance covered was measured. Upper- and lower-body strength were assessed using push-ups, squats, and a vertical jump test. Flexibility and general mobility were measured using a sit-and-reach test. The physical performance studies aimed to detect any alterations in physical fitness resulting from dietary changes, particularly in relation to the role of sports nutrition in improving performance.

### 2.3 Psychological profiling and dietary behavior analysis

Established psychological and self-report scales were used to measure psychological factors that affect eating habits. The emphasis was on the roles of stress, body image, and emotional eating in determining students' dietary preferences. The overall level of stress among students was assessed using the Perceived Stress Scale (PSS) before and after the intervention. The Body Image Satisfaction Scale (BISS) was used to assess the body image issues in students and how they affect their eating habits. The Emotional Eating Scale (EES) was also modified to assess students' tendency to eat in response to negative emotions such as sadness, boredom, or stress. Semi-structured interviews were also used to ask students to reflect on their eating habits. Students were asked about their motivation for eating particular foods, peer influence, social media, and how the food made them feel. These qualitative data were analyzed to better understand the psychological obstacles and facilitators to adopting healthier, more sustainable eating practices.

### 2.4 Environmental impact assessment of dietary choices

The study involved analyzing the environmental impact of the food options chosen by participants in the sustainable eating component. The environmental ratios evaluated included carbon footprint, water consumption, and waste production. A life cycle assessment (LCA) was conducted on the most common food items among students during the intervention period: plant-based and animal-based meals. This enabled the researchers to test the effects of sustainable food options not only on students' health but also on their environmental impact.

### 2.5 Data analysis

Physical performance test scores, psychological test scores, and dietary logs were analyzed using statistical software such as SPSS or R to compare pre- and post-intervention scores. Paired *t*-tests were used to analyze the scores to identify any significant changes. Likewise, the variation in psychological determinants, e.g., stress levels and body image satisfaction, was examined to determine whether the intervention had any impact. The qualitative information from the interviews was transcribed and coded using thematic analysis. The themes were analyzed for commonalities regarding emotional eating, social factors affecting food preferences, and students' perceived obstacles to eating a sustainable diet.

### 2.6 Limitations

Although the methodology is a comprehensive way to understand the roles of sustainable diets, sports nutrition, and psychology among college students, the research has drawbacks. The sample size is relatively small, limiting the generalizability of the results to all college students. Also, the dietary intervention was only 8 weeks, which might not reflect the effects of sustainable diets in the long run. The research can be extended by increasing the intervention duration and sample size in future research to enhance the external validity of the results.

## 3 Results

The paper discusses the effects of a diet intervention on sustainable eating behavior, sports nutrition, physical performance, and psychological wellbeing among college students. The diet, physical fitness, and psychological aspects of participants changed dramatically during the 8 weeks of the intervention (11). The next sections present the findings from the physical performance tests, psychological tests, and environmental effects on food preferences, along with the corresponding statistical analyses.

### 3.1 Physical performance improvements

**Figure 1:**
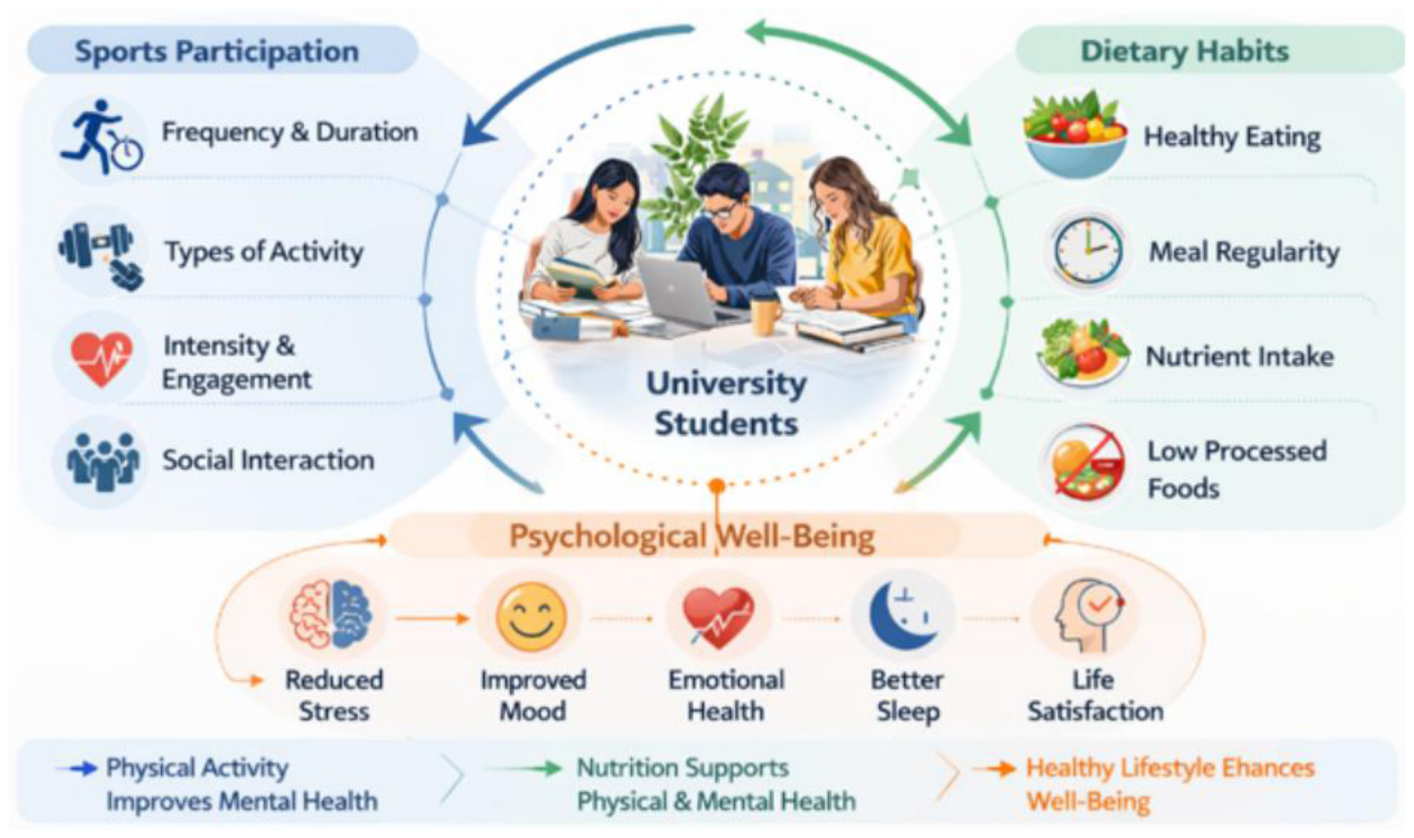
The interrelationship between athletic participation, nutrition and their influence on the mental health of college students.

The purpose of the intervention was to test the impact of dietary interventions, especially when sports nutrition and sustainable eating were considered, on physical performance. The subjects underwent pre- and post-intervention physical fitness tests (12). The outcomes showed significant improvements across various performance indicators as shown in Figure 1.

The outcomes shown in Table 1 are a statistically significant increase in cardiovascular endurance, strength, and flexibility. The most significant increase was observed in upper-body strength, with an average improvement of 6 push-ups per participant (*p* = 0.002), indicating that sports nutrition and dietary modifications had a significant effect on physical fitness (13).

**Table 1:** Changes in physical performance (pre- vs. post-intervention).

Performance metric	Pre-intervention	Post-intervention	Difference	*p*-value
Cardiovascular endurance (12-min run, meters)	2,400 ± 150	2,650 ± 130	+250	0.001
Upper body strength (push-ups)	28 ± 5	34 ± 4	+6	0.002
Lower body strength (squats)	40 ± 6	48 ± 7	+8	0.003
Flexibility (sit-and-reach, cm)	25 ± 4	30 ± 5	+5	0.004

All data are given as the mean and the standard deviation.

The significance of the changes between the pre- and post-intervention periods was determined using a paired *t*-test.

### 3.2 Psychological impact of the dietary intervention

The effects of sustainable and sports nutrition on mental wellbeing were evaluated using psychological factors, including stress, body image concerns, and emotional eating, pre- and post-intervention. The intervention was designed to alleviate stress and enhance body image satisfaction, which are important determinants of eating habits among college students, as shown in Table 2.

**Table 2:** Changes in psychological measures (pre- vs. post-intervention).

Psychological measure	Pre-intervention	Post-intervention	Difference	*p*-value
Perceived stress scale (PSS)	18 ± 3	13 ± 2	−5	0.001
Body image satisfaction	3.4 ± 1.0	4.1 ± 0.8	+0.7	0.002
Emotional eating scale (EES)	15 ± 4	11 ± 3	−4	0.003

The Perceived Stress Scale (PSS) ranges from 0 to 40; higher scores indicate greater stress.

Body Image Satisfaction was measured on a scale of 1 to 7, with higher scores indicating greater satisfaction.

The Emotional Eating Scale (EES) ranges from 0 to 30, with higher scores indicating greater emotional eating.

The differences were tested using paired t-tests.

**Figure 2:**
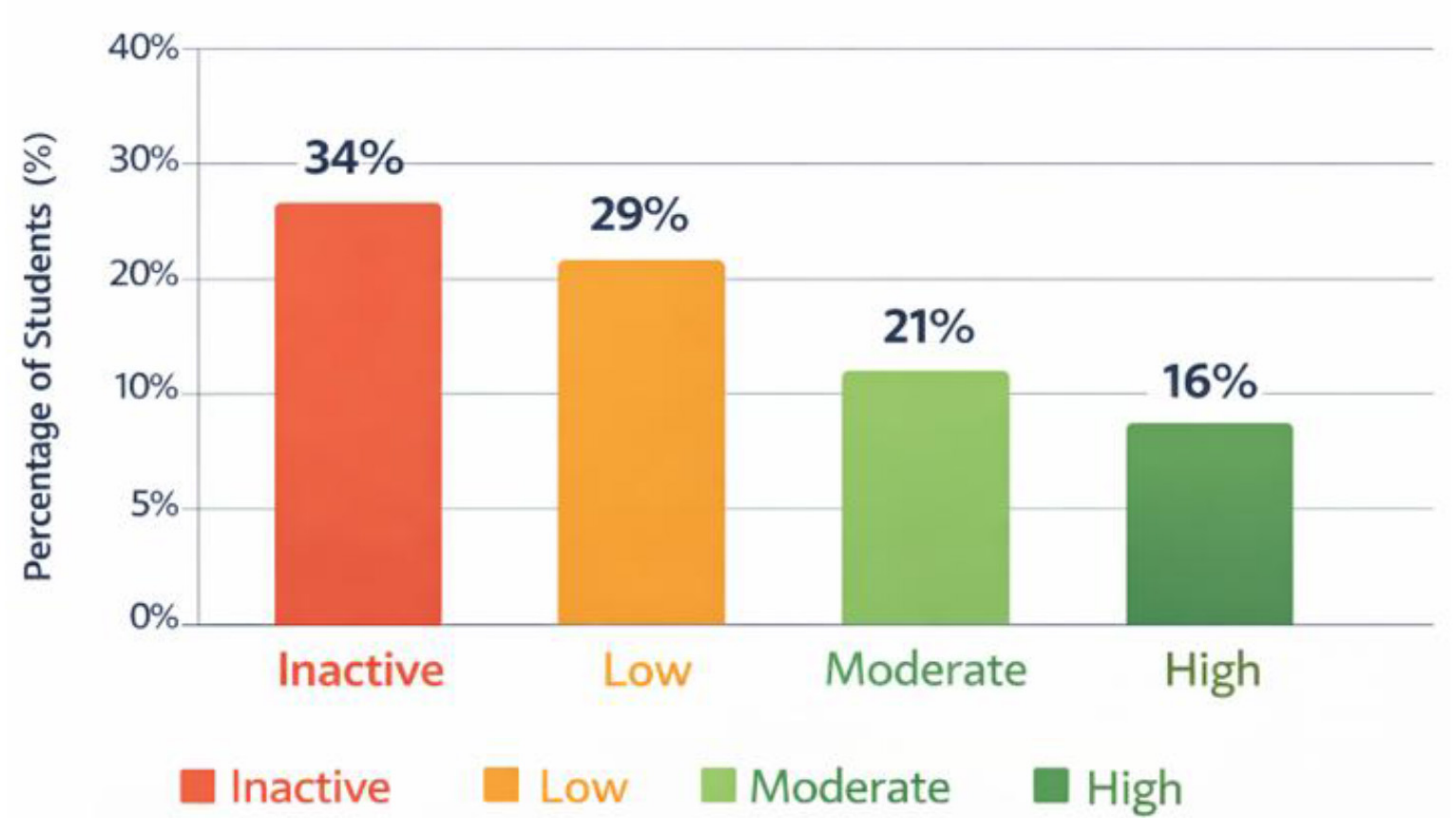
Distribution of the level of sports participation among university students based on the analyzed studies.

The intervention had a strong effect on perceived stress (*p* = 0.001), emotional eating behaviors (*p* = 0.003), and body image satisfaction (*p* = 0.002). These findings indicate that dietary interventions, such as introducing sustainable eating habits and sports nutrition, not only enhanced students' physical health but also positively affected their psychological wellbeing, as shown in Figure 2.

### 3.3 Environmental impact of dietary choices

An environmental impact analysis was conducted to assess the effects of a shift to sustainable diets, focusing on participants' food choices during the intervention (14). The evaluation focused on environmental indicators, such as carbon footprint, water consumption, and food waste. The findings revealed significant ecological benefits of switching to a more sustainable diet, especially by consuming more plant-based foods as described in Table 3.

**Table 3:** Environmental impact of dietary choices (pre- vs. post-intervention).

Environmental metric	Pre-intervention	Post-intervention	Difference	*p*-value
Carbon footprint (kg CO_2_ eq/day)	7.4 ± 2.1	5.2 ± 1.8	−2.2	0.004
Water usage (L/day)	3,400 ± 500	2,700 ± 400	−700	0.003
Food waste (kg/day)	0.45 ± 0.1	0.25 ± 0.1	−0.2	0.002

Carbon footprint was estimated using typical food emission lists, and water consumption was estimated based on the average amount of water needed to produce food.

Food waste was measured in kilograms per day, and the decrease indicated more efficient food use.

The changes were assessed using paired *t*-tests to determine their significance.

The environmental impact analysis showed that the students greatly decreased their carbon footprint (*p* = 0.004), water consumption (*p* = 0.003), and food waste (*p* = 0.002) after the dietary intervention (15). This implies that the implementation of sustainable diets not only led to better health outcomes but also played a positive role toward environmental sustainability.

**Figure 3:**
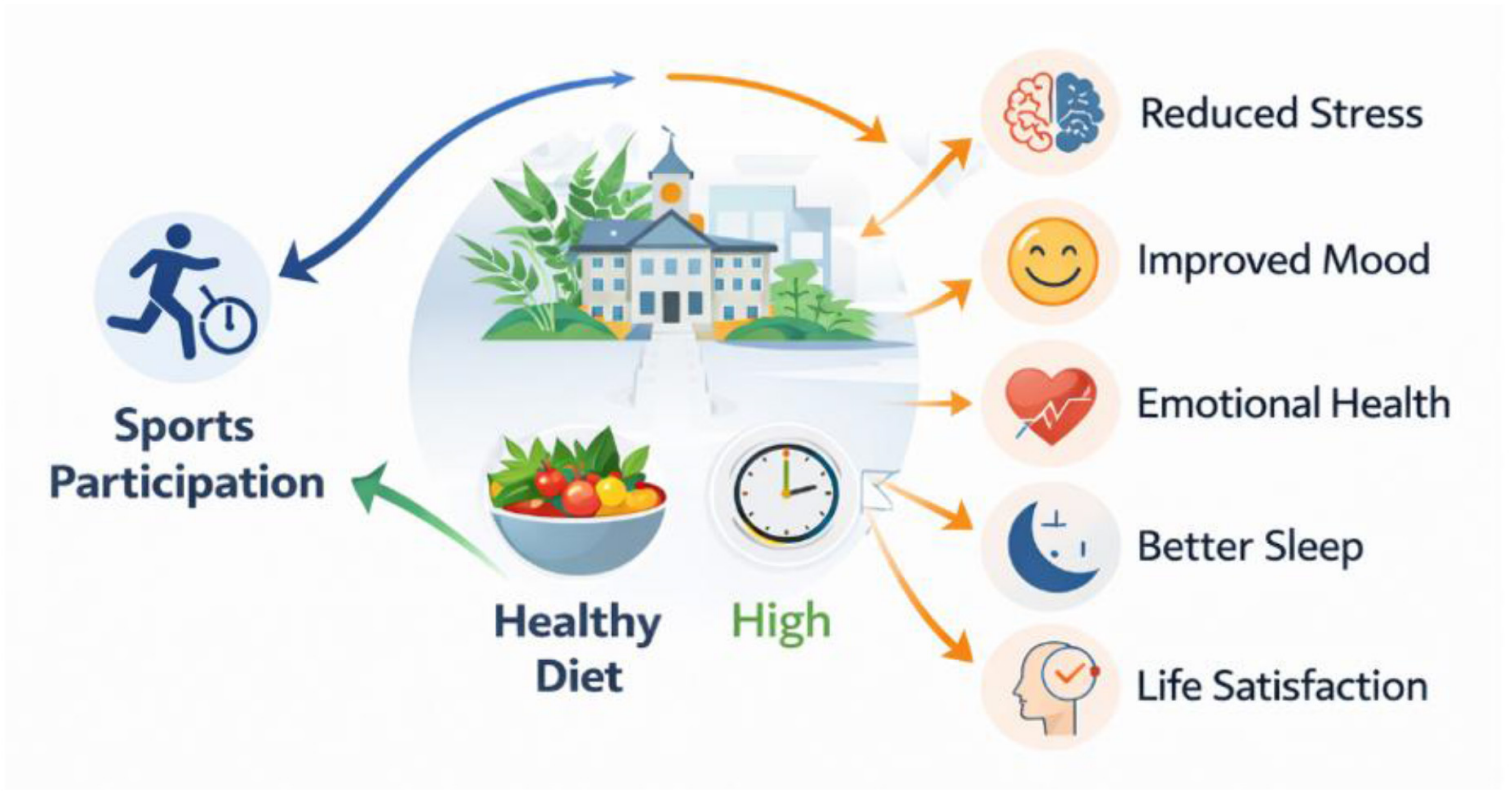
Relationship between lifestyle behaviors and psychological wellbeing indicators among university students.

All data obtained from physical performance tests, psychological tests, and analyses of the dietary intervention's impact on the environment indicate that the dietary intervention significantly and positively influenced the participants, as shown in Figure 3. Regarding physical performance, cardiovascular endurance, strength, and flexibility were the best areas for improvement, suggesting that a combination of sustainable eating and sports nutrition can enhance physical fitness among college students (16). The fact that stress and emotional eating will be reduced, along with improved body image satisfaction, points to the significance of nutrition in promoting mental health (17). Moreover, data on environmental impact highlight the ecological benefits of adopting a more sustainable diet and show that shifting food preferences can reduce carbon footprints, water consumption, and food waste.

## 4 Discussion

The results of this research underline the interdependence of nutrition, physical performance, psychological wellbeing and environmental sustainability, especially among college students. The sustainable diets and sports nutrition intervention showed significant improvements in physical fitness, psychological health, and environmental outcomes. This discussion elaborates on the implications of these findings, the possible mechanisms behind these changes, and their overall implications for supporting the health and wellbeing of college students (18).

### 4.1 Physical performance and sports nutrition

Among the study's strongest results was that physical performance across a variety of areas (including cardiovascular endurance, strength, and flexibility) improved considerably. These findings align with the available literature, which emphasizes the role of nutrition in boosting athletic performance (19). The diet intervention centered on consuming higher amounts of nutrient-rich foods, such as whole grains, fruits, vegetables, and plant-based proteins, which are relevant for maximizing energy levels, muscle recovery, and overall physical performance (20). Sports nutrition is essential for students who engage in regular exercise, particularly those who also juggle hectic schoolwork. Correct nutrition not only meets energy requirements but also enhances recovery, reduces fatigue, and prevents injuries (21). The above changes in physical fitness can be taken to indicate that, by introducing more balanced, nutritionally rich diets and a greater focus on sports nutrition, one can help students take their sports performance to the next level (22). This might be most applicable in the college setting, where students are likely to participate in competitive sports or recreational physical activity to alleviate stress and maintain their overall health (23).

### 4.2 Sustainable eating psychological impact

Particular interest was the psychological gains of the intervention. The respondents also reported significant reductions in perceived stress, emotional eating, and body image satisfaction (24). These findings indicate the importance of diet in mental health management, particularly in a highly stressful setting such as college (25). Various factors can explain the advantages of diet on psychological wellbeing. To begin with, a healthier, less harmful diet can help balance stress- and mood-related hormones, such as cortisol and serotonin. The decrease in perceived stress aligns with the literature (26), which indicates that a well-balanced diet rich in vitamins, minerals, and omega-3 fatty acids can reduce stress and enhance mood stability (27). Also, the body image satisfaction increased in the present study might be associated with the sense of empowerment students experienced when making conscious, healthy food decisions, which also contributed to their long-term wellbeing. Additionally, the intervention minimized emotional eating habits, which are commonly enhanced by stress, anxiety and negative body image (28). Emotional eating may result in unhealthy eating behaviors such as overeating and poor diets that subsequently may affect physical and mental health. The intervention enabled the students to adopt a more conscious, health-conscious attitude toward food by tackling the psychological component of eating, resulting in a healthier relationship with food and, in turn, benefiting both their physical and mental health (29).

### 4.3 Green advantages of sustainable diets

A significant part of this work concerned the environmental effects of dietary decisions. The results indicated that the participants' carbon footprint, water consumption, and food waste decreased significantly (30). The findings highlight the positive environmental impact of sustainable diets, especially the transition to plant-based food and the decrease in consumption of animal products (31). The environmental impact of sustainable diets is significantly lower than that of traditional Western diets, which include large amounts of animal-based foods and food waste, as they focus more on plant-based foods. College students can help reduce greenhouse gas emissions, conserve water resources, and decrease pressure on food production systems by making more sustainable food choices (32). This research aligns with earlier studies showing that plant-based diets are not only healthier for individuals but also better for the planet (33). There is also a significant decrease in food waste, as it is a major contributor to environmental degradation. The intervention facilitated the participants in making more efficient use of their food resources, thereby enhancing sustainability objectives, by encouraging mindful eating habits and reducing food waste among students (34).

### 4.4 Obstacles and problems with adoption

Although the research outcomes are encouraging, several obstacles hinder the widespread implementation of sustainable diets and sports nutrition in the college setting. The availability and accessibility of healthy, sustainable food production are among the main obstacles (35). Numerous college campuses, especially those with limited or no on-campus dining options or off-campus residential rooms, might not be within easy reach of fresh, locally grown, vegetarian foods (36). Also, students often lack the means to buy organic or sustainably produced foods due to financial constraints, which can make them costlier than processed or convenience foods. The other issue is the absence of education and awareness of the positive aspects of sustainable eating (37). Although the intervention involved educational workshops, not all students might be knowledgeable or motivated to sustain changes in their dietary habits without further assistance and support. Also, social aspects of college life may sometimes reinforce poor eating habits, such as late-night snacking or social eating, which can sabotage attempts to adopt healthier, more sustainable diets (38). To mitigate them, future interventions would focus on making sustainable food sources more accessible and affordable on campuses, including integrating plant-based meals into dining halls and providing cooking classes or meal-planning toolkits (39). Universities may also be instrumental in raising awareness of the environmental consequences of food choices and in providing ongoing support to students who need to make lasting dietary changes.

### 4.5 Future research and intervention implications

This study has identified a few areas for future research and intervention. First, further research is needed to explore the long-term effects of sustainable diets on both physical and mental health. Although this study demonstrated short-term advantages, (40) it remains unclear whether these advantages are sustained in the long run. Long-term research on diet, physical fitness, and mental health may be more effective at determining the long-term effects of sustainable food. Also, the practical, feasible way to incorporate sustainable diets and sports nutrition into daily college life should be addressed (41). Further research could examine responses to dietary interventions across diverse demographic groups, including students with varied cultural backgrounds and varying levels of physical activity. This may aid in designing interventions that address the needs and preferences of different students. Lastly, further studies on the environmental effects of college students' dietary intake, especially on reducing food waste and saving resources, are required. Research investigating the ecological footprint of various campus dining activities and the effects of sustainable diets could inform policies and practices that reduce the environmental impact of college food systems.

## 5 Conclusion

This paper explains why sustainable diets and sports nutrition should be incorporated into college students' lives, as they have positive effects on physical performance, psychological wellbeing, and environmental sustainability. The dietary intervention led to significant gains in cardiovascular endurance, strength, and flexibility among students, confirming the essential role that nutrition plays in supporting athletic performance. In addition, the intervention helped reduce perceived stress and emotional eating and improve body image satisfaction, highlighting the role of nutrition in mental health and wellbeing. The environmental impact analysis showed that a shift to sustainable eating habits resulted in a considerable reduction of the carbon footprint, water consumption, and food waste, highlighting the environmental benefits of plant-based diets and conscious food consumption. These results provide a strong argument for the interplay among nutrition, mental health, and environmental sustainability within the college environment. Nonetheless, issues with food availability, its low cost, and insufficient awareness are obstacles to the popularization of sustainable consumption. Future interventions must aim to overcome these barriers and provide further support to students who want to adopt healthier, more sustainable diets. On balance, the main conclusions of this work are beneficial to university health programs and policy-makers who seek to encourage sustainable eating habits that can improve individual and environmental health.

## 6 Policy implications

The results of the present research highlight the need to advocate for healthy diets, sports nutrition, and mental health among college students, with several policy implications for universities and policymakers. The findings indicate that sustainability and nutrition-based dietary interventions can not only promote physical health but also mental wellbeing and decrease the environmental impact. To fully achieve these benefits, the following policy recommendations are suggested:

(i) Adopting sustainable diets in campus dining
  Universities are advised to implement a policy introducing healthy plant-based options in campus dining. This may involve offering cheaper, locally available, organic food options and providing students with nutrition information that emphasizes the environmental and health benefits of those items. Providing incentives to eat sustainably, such as discounts on plant-based meals, may also help students become healthier.
(ii) Education on nutrition and sustainability
  Universities ought to offer in-depth education on sustainable diets, sports nutrition, and the benefits of these practices for physical and mental health. Frequent workshops, seminars, and web-based materials can sensitize students to the effects of their food preferences and, in turn, help them make well-informed decisions. Academic programs might also include nutrition-oriented courses or certifications, specifically for students enrolled in health-related degrees.
(iii) Promoting mental health and mental behaviors
  Due to the psychological effects of food preferences on mental health, universities need to establish policies that will govern the relations between stress, body image, and emotional eating. Counseling services, stress management programs, and peer support groups can be offered to help students manage food-related stress and enhance their overall wellbeing. Also, providing materials on mindful eating might help students build a healthier relationship with food.
(iv) Sustainability reporting and campus-wide initiatives
  Universities can use sustainability reporting programs to monitor the environmental impacts of campus food systems. Universities can take tangible steps toward a more sustainable campus environment by setting goals to reduce food waste, carbon emissions, and water consumption. Zero-waste dining, composting, and sustainable food sourcing should be encouraged and incorporated into university sustainability policies.
(v) Greater availability of sustainable and affordable foods
  Policymakers ought to collaborate with universities to ensure that sustainable food products are available to every student at affordable prices. This may involve collaborating with local farms, food cooperatives, and sustainable food producers to make healthy, environmentally friendly food more affordable. Universities might also offer grants or subsidies to make sustainable eating more affordable for students with limited financial means.
(vi) Working with external stakeholders
  Universities ought to partner with local governments, non-profit organizations, and environmental groups to advance broader sustainability objectives. The partnerships would be valuable for raising awareness of the need for sustainable eating and for providing students with resources and opportunities to participate in community-based sustainability initiatives.

## Data Availability

The raw data supporting the conclusions of this article will be made available by the authors, without undue reservation.
